# Validity of a 5-minute focused echocardiography with A-F mnemonic performed by non-echocardiographers in the management of patients with acute chest pain

**DOI:** 10.1186/s12947-015-0010-y

**Published:** 2015-03-26

**Authors:** Dorota Sobczyk, Krzysztof Nycz, Pawel Andruszkiewicz

**Affiliations:** The Department of Interventional Cardiology, John Paul 2nd Hospital, Pradnicka 80, 31 202 Cracow, Poland; 2nd Clinic of Anaesthesiology and Intensive Care, Warsaw Medical University, Warszawa, Poland

**Keywords:** Acute coronary syndrome, Emergency room, Focused echocardiography, Emergency echocardiography, A-F mnemonic

## Abstract

**Study objective:**

To validate the practicality of focused echocardiography with A-F mnemonic performed by non-specialists in patients with suspected acute coronary syndrome (ACS).

**Design:**

This prospective observational study was conducted in the Emergency Room within 12 months period. Study population consisted of consecutive patients with preliminary diagnosis of an ACS.

The following data were analyzed: demographics, clinical condition, medical history, ECG, transthoracic echocardiography (TTE) and levels of cardiac necrotic markers. TTE was performed within the first 15 minutes after the admission by the resident on-call. TTE images were interpreted and reported with mnemonic A-F. All studies were recorded and reviewed within 24 hours by the cardiologist.

**Results:**

1312 consecutive patients were enrolled to the study. TTE with A-F mnemonic revealed: RWMAs in 82,87% patients with confirmed ACS, other significant cardiac pathologies were found in 2,21% in ACS and 46,52% in non-ACS groups respectively. On the basis of these findings, 20 (1,92%) ACS and 29 (10,62%) non-ACS group patients underwent target operative treatment. Survey showed that both echocardiographic image acquisition and its interpretation with A-F mnemonic, took less than 5 minutes in 95% of cases. Residents found A-F mnemonic algorithm simple and useful. No differences were found in key findings between TTE performed by resident and the cardiologist.

**Conclusion:**

Focused echocardiography with A-F mnemonic allows both confirmation of acute myocardial ischemia and detection of the other life-threatening cardiac conditions resulting in proper bedside decision of directed treatment. Mnemonic based TTE enables reliable examination by properly trained residents.

## Introduction

The workup of chest pain management (including suspected ACS) includes: clinical presentation, medical history, physical examination, risk stratification, laboratory tests, and non-invasive imaging [1-4]. Among non-invasive imaging techniques transthoracic echocardiography (TTE) plays a pivotal role in acute setting [5-7]. However, full echocardiographic examination is time-consuming. It is usually performed by a cardiologist with an expertise in clinical echocardiography who is not easily available at the time of emergency admission. In such situation when time is an important issue, introduction of a focused, simplified echocardiographic scheme for primary screening and reporting is desirable. Such algorithm should be easy to perform, reproducible, directed at the most common life-threatening pathologies and possible to learn by a non-specialist during a short training. The A-F mnemonic meets all of these criteria and could be easy implemented in the emergency room settings [8].

### Study objective

The purpose of the present study was to validate the usefulness and practicality of focused transthoracic echocardiography with A-F mnemonic, performed by non-echocardiographers in patients with acute chest pain.

## Material and methods

The observational study was approved by the Local Ethical Committee of John Paul 2nd Hospital in Cracow (ref. number 792) and informed consent was obtained from all study participants.

The study was conducted in the Emergency Room of the Interventional Cardiology Department of John Paul 2nd Hospital in Cracow, within 12 months period (January 2013 to January 2014).

We prospectively enrolled all patients admitted to the Emergency Room with preliminary diagnosis of an acute coronary syndrome made by the pre-hospital health care providers. The diagnosis was based on typical clinical symptoms and ECG findings: new or presumed new significant ST-segment-T-wave (ST-T) changes, new left bundle branch block (LBBB) or development of pathological Q waves. Exclusion criteria were: age < 18 years and difficult acoustic window resulting in inability to obtain interpretable ultrasound images. 59 patients (59/1371, 4.3%) were excluded from final analysis because of difficult acoustic window and un-interpretable ultrasound images.

14 residents (at least after the completion of third year of training) were included in the study. All of them had basic skills in TTE and performed at least 75 examinations before participation in the study. Additionally, they underwent a minimum of 30-minutes didactic and 1,5-hour structured hands-on dedicated practice with the A-F examination scheme.

In the Emergency Room, clinical condition was assessed according to both Canadian Cardiovascular Society (CCS) and New York Heart Association (NYHA) classifications. Prior myocardial infarction, coronary revascularization and pre-hospital cardiac arrest, were noted. In order to confirm the diagnosis of ACS, further investigations were instituted including: ECG, transthoracic echocardiography (TTE) and blood tests for measurement of biomarkers for myocardial damage.

Electrocardiogram (ECG) was performed within 5 minutes after the admission to the Emergency Room and repeated in 15-minutes intervals in symptomatic patients with an initial non-diagnostic ECG and during every recurrence of symptoms. The ECG recording included: standard 12-leads, right precordial leads (V3R, V4R) and posterior leads (V7-V9) when appropriate.

According to newly introduced institutional fast-track protocol, transthoracic bedside echocardiography was performed within the first 15 minutes after patient’s presentation, by the resident on-call. Duration of the examination was recorded and usefulness of the mnemonic was assessed by the physicians. TTE images were interpreted with the simplified mnemonic A-F, introduced in our Emergency Room in order to standardize bedside cardiac examination. In A-F mnemonic, consecutive letters of an alphabet represent a particular anatomical structure or measure of cardiac function: A-aorta, B-both ventricles, C-contractility, D-dimensions, E-effusion, F-further abnormalities (Table 1). The examinations were conducted with portable ultrasound system equipped with a 1–5 MHz transthoracic phased-array transducer (Vivid I, GE Healthcare, USA, and CX 50, Philips, Eindhoven, Netherlands). Heart was visualized in 5 basic echocardiographic views: parasternal long axis, parasternal short axis, apical four-chamber, apical two-chamber and subcostal. All studies were recorded as digital 10-seconds video clips and reviewed within 24 hours by the consultant cardiologist unaware of the resident’s conclusions. For the purpose of the study, the cardiologist was asked to summarize his/her findings using AF-mnemonic tool structure and if necessary to add important information extending beyond this scheme. Both summaries were confronted by independent researcher (experienced cardiologist-echocardiographer) during the analysis and the decision was made if any discrepancies between the two diagnoses might have had any impact for the choice of treatment and the final outcome.

**Table 1:** **Description of the mnemonic A-F algorithm**

**Letter**	**Description**	**Question**	**Possible diagnosis (if the answer is YES)**
A	Aorta	Is aortic root dilated?	Aortic root dilatation/aneurysm
		Is proximal aortic diameter > 4 cm?	Ascending aortic dilatation/aneurysm
		Is dissection flap seen?	Aortic dissection
B	Both ventricles	Is there RV overload present?	Pulmonary embolism
		RV/LV > 1	Pulmonary hypertension
		D-sign	RV infarction
C	Contractility	Is LV contractility impaired?	
		Depressed global systolic function	LV heart failure
		Regional wall motion abnormalities	Acute myocardial infarction
		Is RV contractility impaired?	
		Depressed global systolic function	RV heart failure; RV infarction; pulmonary embolism
D	Dimensions	Are there any abnormal dimensions?	
		Ascending aorta > 4 cm	Aortic dilatation/aneurysm (look for aortic dissection)
		LV end-diastolic dimension > 6 cm	LV dilatation (assess global LV function)
		RV end-diastolic dimension > 4.2 cm	RV dilatation (look for the RV overload)
		LA anteroposterior dimension > 4.5 cm	LA dilatation
		RA major > 5.4 cm and/or minor dimension > 4.4 cm	RA dilatation (look for RV overload)
E	Effusion	Is pericardial effusion present?	Pericardial effusion
		Are there any signs of cardiac tamponade?	Cardiac tamponade
		RA end-systolic or diastolic collapse	
		RV diastolic collapse	
		Vena cava plethora	
		Is there pleural effusion?	Pleural effusion
F	Further abnormalities	Any other abnormal findings not listed above?	

From: Sobczyk and Andruszkiewicz P [8].

Blood samples were collected for plasma levels of biomarkers of myocardial damage: creatine kinase (CK), cardiac isoenzyme of creatine kinase (CK-MB) and high sensitivity troponin T (hsTnT), at the time of admission and, if necessary, by repeated measurements every 3–6 hours within the first day of hospitalization. Blood tests were assayed by routine automated laboratory techniques (Cobas System 6000, Roche Diagnostics GmbH, Manheim, Germany). All biochemical analyses were performed in the central hospital laboratory, certified with cardiac and clinical chemistry program by RIQAS (Randox International Quality Assessment Scheme, UK).

**Figure 1:**
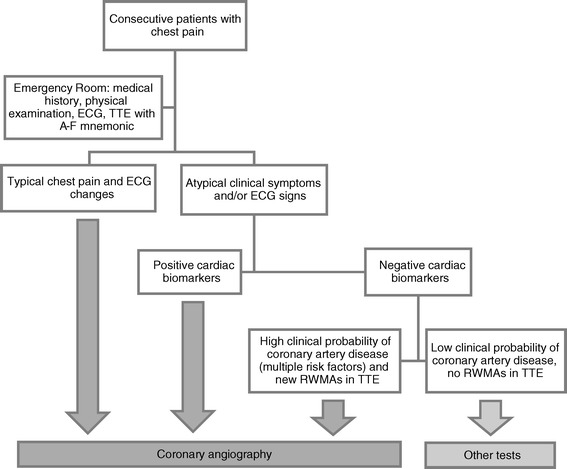
**Flow chart of patients in the study.**

Coronary angiogram was performed in the following groups of patients: with typical chest pain and typical ischemic ECG changes (as stated above), regardless of cardiac biomarkers on admission; with atypical clinical presentation (atypical chest pain, non-diagnostic ECG) but with elevated cardiac biomarkers on admission and/or typical evolution (rise or fall) of biomarkers in following 3–6 hours; with atypical clinical presentation (atypical chest pain, non-diagnostic ECG) and negative cardiac biomarkers, but with multiple risk factors (e.g. hypertension, dyslipidemia, diabetes, previous MI, previous revascularization, positive family history) and new or presumed new regional wall motion abnormalities in TTE on admission (performed according to A-F mnemonic) (Figure 1).

Medical data were analyzed independently by the authors and in each case a consensus was achieved. No ambiguous echocardiographic examinations were recorded, all results were clearly stated in a dichotomic manner: echocardiographic abnormality present or not.

Statistical analysis was performed using STATISTICA v 8.0 software. Numerical data were expressed as mean values ± SD.

## Results

1312 consecutive adult patients (858 male, age 21–98 years, mean age 67 ± 12,32 years) were enrolled to the study. Baseline demographic and clinical data are shown in Table 2. Echocardiographic findings are summarized in Table 3.

**Table 2:** **Demographic and clinical characteristics of the study population**

**Characteristic**	**Value**
Age (years)	67 ± 12, 32
Male sex, n (%)	858 (65,39)
CCS class	3,38 ± 0,73
NYHA class	1,64 ± 0,97
Concomitant diagnosis, n (%):	
Prior myocardial infarction	268 (20,43)
Prior PCI	257 (19,59)
Prior CABG	70 (5,34)
Prior AVR	12 (0,91)
Pre-hospital cardiac arrest, n (%)	76 (5,79)
ECG changes at admission, n (%):	
ST depression	742 (56,55)
ST elevation	490 (37,35)
Q/QS complex	213 (16,23)
Negative T-waves	161 (12,27)
LBBB	49 (3,73)
Other	167 (12,73)
hs TnT (ng/ml)	0,41 ± 0,86
CPK (U/l)	452,96 ± 2256,02
CM-MB (U/l)	40,86 ± 54,23

PCI-percutaneous coronary intervention; CABG-coronary artery bypass grafting; AVR-aortic valve replacement; LBBB-left bundle branch block; hs TnT-high sensitivity troponin T; CPK-creatine kinase; CK-MB-cardiac isoenzyme of creatine kinase.

**Table 3:** **Echocardiographic findings in study population**

**Echocardiographic mnemonic**	**Findings**	**Value**
A (aorta)	Aortic dilatation, n (%)	62 (4,73)
	Aortic dissection, n (%)	5 (0,38)
B (both ventricles)	Right ventricular dilatation, n (%)	46 (3,51)
	Right ventricular overload, n (%)	20 (1,52)
C (contractility)	Regional wall motion disturbances, n (%)	921 (70,19)
	LVEF (%)	46,569 ± 13,41
	LVEF ≤ 30%, n (%)	199 (15,17)
D (dimensions)	Abnormal heart dimensions, n (%)	325 (24,77)
	Left ventricular dilatation, n (%)	71 (5,41)
	Left ventricular hypertrophy, n (%)	145 (11,05)
	Left and right atrial dilatation, n (%)	104 (7,97)
	Right ventricular dilatation, n (%)	54 (4,12)
E (effusion)	Pericardial or pleural effusion, n (%)	61 (4,65)
	Pericardial effusion, n (%)	48 (3,66)
	Cardiac tamponade, n (%)	3 (0,23%)
	Pleural effusion, n (%)	18 (1,37)
F (further abnormalities)	Further abnormalities, n (%)	242 (18,45)
	Mitral valve abnormalities, n (%)	126 (9,60)
	Aortic valve abnormalities, n (%)	77 (5,87)
	Other, n (%)	78 (5,95)
A + B + E + F	Any abnormality	319 (24,31)
Normal study	No abnormalities	74 (5,64)

LVEF-left ventricular ejection fraction.

Chest pain was present in 98,1% of patients, with 51,68% of patients in 4 CCS class. Dyspnea was observed in 36,28% of patients, with 8,69% of patients in NYHA 4 class. ECG on admission revealed ST-depression in 56,55% and ST-elevation in 37,35% of patients (Table 2). Troponin T levels were higher than 99th percentile of upper range limit (URL) in 87,42% of patients, exceeding three times URL in 64,94% of cases (Table 2). Coronary angiography was performed in 95,05% of patients. 75,3% of them had subsequent coronary revascularization (PCI or CABG). The final diagnosis of myocardial infarction (with or without persistent ST-segment elevation), unstable angina (UA) or Takotsubo cardiomyopathy, was determined according to the recent clinical guidelines (the group with confirmed ACS). Final ACS diagnosis was confirmed in 79,19% of enrolled patients.

Echocardiography performed on admission showed regional wall motion abnormalities (RWMAs), with normal wall thickness and no features of post-infarction scar, in 99,5% of patients with STEMI and 78,5% with NSTEMI, and it was considered an echocardiographic finding enhancing the probability of ACS. Coexisting cardiac pathology (apart from ACS) was revealed in 23 patients (2,21%) with confirmed ACS. In 20 of them (86,96%), with significant structural disease (at least moderate aortic stenosis, severe mitral regurgitation, post-infarction VSD), TTE was a decisive diagnostic tool determining the choice of a treatment. After coronary angiography, all these patients underwent urgent coronary artery bypass grafting with additional target procedure: aortic valve replacement, mitral valve annuloplasty or VSD closure.

In patients in whom the initial suspicion of ACS was not finally confirmed, the final diagnosis was established based on echocardiography alone or additional diagnostic tests (blood tests, chest X-ray, computed tomography). In 127 patients (46,52%) from this group, clinically significant echocardiographic abnormalities were found during the AF-mnemonic - based TTE exam (Table 4). On the basis of these findings, 29 patients (10,62%) underwent invasive treatment: aortic valve replacement, mitral annuloplasty, aortic alloplasty, decompression of tamponade, VSD closure, fibrinolysis, removal of infected pacemaker electrode.

**Table 4:** **Final diagnosis of patients admitted with the primary diagnosis of ACS with the prevalence of typical echocardiographic findings reported by limited TTE with A-F mnemonic**

**Final diagnosis**	**Number of patients (%)**	**Echocardiographic findings**	**Echocardiographic diagnosis, n (%)**
Confirmed ACS	1039 (79,19)	RWMA	861 (82,87)
NSTEMI	543 (52,26)	RWMA	426 (78,45)
STEMI	387 (37,25)	RWMA	385 (99,48)
UA	92 (8,85)	RWMA	33 (35,87)
Takotsubo cardiomyopathy	17 (1,65)	RWMA, apical ballooning	17 (100)
Adjective diagnosis in confirmed ACS	23 (2,21)	listed below	23 (100)
Moderate/severe aortic stenosis	16 (1,54)	AV calcification	16 (100)
Severe mitral regurgitation	3 (0,29)	MR jet	3 (100)
Decompensated CHF	3 (0,29)	dilated LV, LA, small pericardial/pleural effusion, low LVEF	3 (100)
Ventricular septal defect	1 (0,09)	IVS interruption	1 (100)
Non-confirmed ACS	273 (20,81)	any abnormality	151 (55,31)
Decompensated CHF	51 (18,68)	dilated LV, LA, small pericardial/pleural effusion, low LVEF	51 (100)
Stable CAD	35 (12,92)	RWMA	0 (0)
Acute pulmonary embolism	21 (7,69)	RV overload	20 (95,27)
Aortic valve disease	15 (5,49)	AV calcification	15 (100)
Myocarditis	10 (3,66)	moderately impaired global LV contractility	8 (80)
Tachycardia (AF, SVT)	10 (3,66)	atrial dilatation	8 (80)
Pericarditis	8 (2,93)	pericardial effusion, thickened pericardium	8 (100)
Prinzmetal angina	8 (2,93)	none	8 (100)
Pneumonia	6 (2,19)	small pleural effusion	4 (66,666)
Mitral valve disease	5 (1,83)	MR jet	5 (100)
Aortic dissection	5 (1,83)	dilated aorta, dissection flap	5 (100)
Hypertensive crisis	6 (2,19)	LVH	6 (100)
Exacerbated COPD	4 (1,47)	RV dilatation	4 (100)
HOCM	4 (1,47)	severe LVH, LVOTO	4 (100)
Cardiac tamponade	3 (1,09)	pericardial effusion, RA and RV collapse	3 (100)
Ventricular septal defect	2 (0,73)	IVS interruption	2 (100)
Hypovelemic shock	1 (0,37)	small LV, IVC < 1 cm	1 (100)
Infective endocarditis associated with pacemaker electrode	1 (0,37)	abnormal structures on the pacemaker electrode	1 (100)
Perivalvular leak in AVR	1 (0,37)	perivalvular jet	1 (100)
Lung tumor	1 (0,37)	none	1 (100)
Acute pancreatitis	1 (0,37)	none	1 (100)
Exacerbated polimyositis	1 (0,37)	none	1 (100)
Other	71 (26,01)	no abnormal findings	71 (100)

ACS-acute coronary syndrome; NSTEMI-non-ST-elevation myocardial infarction; STEMI-ST-elevation myocardial infarction; UA-unstable angina; RWMAs-regional wall motion abnormalities; AV-aortic valve; MR-mitral regurgitation; LA-left atrium; LV-left ventricle; IVS-inteventricular septum; RV-right ventricle; RA-right atrium; IVC-inferior vena cava; CHF-chronic heart failure; COPD-chronic obstructive pulmonary disease; AVR-aortic valve replacement; HOCM-hypertrophic obstructive cardiomyopathy; LVH-left ventricular hypertrophy; LVOTO-left ventricular outflow tract obstruction.

Survey of the residents involved in the study, showed that focused echocardiographic examination and its interpretation based on A-F mnemonic, took less than 5 minutes in 95% of cases. All of them found A-F mnemonic algorithm very useful in sticking to the examination structure and avoiding omissions of the important findings. No differences were found in key findings and conclusions between TTE performed by the resident on-call and the cardiologist (Table 5). In 242/1312 (18,45%) cases cardiologists reported extra findings extending beyond the A-F scheme (mild mitral valve regurgitation, mild or moderate tricuspid valve regurgitation, mild left atrial enlargement). According to the cardiologist in charge of the study none of these, however, had any impact on the choice of the instituted treatment.

**Table 5:** **Comparison of A-F-based echocardiography performed by residents on call and examination reported by cardiologist**

**A-F mnemonic**	**Findings**	**Concordance between the reports (%)**
A	Aortic dilatation	100
A	Intimal flap	100
B	RV dilatation	100
B	RV overload (RV > LV)	100
C	RWMAs	100
C	Apical balooning	100
C	Severely depressed global LV function	100
D	Abnormal heart dimensions (mild left atrial dimension)	57,32
E	Pericardial effusion	100
E	Pleural effusion	100
F	Further abnormalities (mild mitral regurgitation, mild/moderate tricuspid regurgitation)	70,76

RV-right ventricular; RWMAs-regional wall motion abnormalities; LV-left ventricular.

## Discussion

According to recent clinical guidelines echocardiography plays an important role as a first-line emergency diagnostic tool in patients with chest pain (including suspected ACS) [2-5]. It is a quick and safe method to establish dynamic cardiac anatomy and pathophysiology. Echocardiographic evidence of new regional wall motion abnormalities is one of the diagnostic criteria for an acute myocardial infarction (when combined with detection of rise and/or fall of cardiac biomarkers) [2] whereas impaired left ventricular global systolic function is a factor related with a poor prognosis. Moreover, TTE can facilitate differential diagnosis of various diseases related with chest pain [2-4,6,7,9-13]. Several life-threatening conditions (acute aortic dissection, acute pulmonary embolism, cardiac tamponade, severe valvular disease or hypertrophic cardiomyopathy) requiring urgent treatment, can be identified with this bedside tool [9-13].

Although some might argue that TTE has no added value in the non-shocked patient, all recent clinical guidelines for diagnosis and management of myocardial infarction, emphasize the role of TTE as the initial imaging modality in patients with acute chest pain [2-4,12-14]. Everyday emergency practice in our center also confirms the importance of this diagnostic procedure. We have observed numerous patients with typical chest pain and ST-segment changes (also ST elevation) caused by other than ACS, life-threatening conditions that could have been detected by focused TTE, i.e. acute aortic dissection, cardiac tamponade or acute pulmonary embolism. In this subset of patients, immediate coronary angiography may expose them to an unnecessary risk (i.e. catheterization of false lumen in patients with acute proximal aortic dissection) and finally delay appropriate treatment. That is why we believe that echocardiography should be routinely available in Emergency/Admission Room and implemented in all patients with suspected ACS (if it is possible without delay of target treatment). Based on both current recommendations and own experience, focused TTE with A-F mnemonic was introduced in our hospital as an integral and mandatory part of an initial evaluation of all patients admitted due to chest pain/suspected ACS. It must be highlighted that bedside TTE with A-F mnemonic is not equivalent and does not replace the comprehensive echocardiographic examination, performed as recommended by the guidelines.

Performing TTE should not delay direct treatment of suspected ACS. In all patients with typical clinical manifestation and ST elevation on ECG, emergency coronary angiography and subsequent revascularization should be initiated as soon as possible. However, limited bedside TTE usually does not take more than five minutes and may be performed simultaneously with other procedures on admission (ECG recording, collecting blood samples or preparation for coronary angiography). High prevalence of other cardiac and non-cardiac life-threatening conditions mimicking the clinical presentation of ACS, justifies the implementation of additional imaging tests in the management of patients with suspected ACS. In our study population, final ACS diagnosis was confirmed in 79,19% of enrolled patients.

Correct and quick diagnosis of a suspected acute coronary syndrome at admission determines outcome of the treatment. However, unquestionable value of TTE may be limited by the lack of skill and competency in the physicians. The full echocardiographic examination seems to be too complicated, extensive and time-consuming and some important pathologies may be overlooked and omitted especially when distracting abnormalities are present. Thus echocardiographic diagnosis of ACS in an Emergency Room should concentrate on: evaluation of presence of new RWMAs; global left ventricular systolic function assessment (as a predictor of outcome); and identification of other pathologies leading to chest pain or dyspnea.

In order to improve the standard of care of patients with suspected ACS in our Emergency Room, we decided to introduce a new fast-track protocol based on TTE performed by the residents on-call. Our present study confirmed the utility of early simplified TTE in ACS suspected patients. RWMAs were present in 82, 87% of patients with confirmed ACS (with almost 100% prevalence in STEMI and Takotsubo cardiomyopathy). The study showed that new RWMAs on echocardiography strongly supported ACS diagnosis and was a deciding factor for a further coronary angiography in patients with atypical clinical symptoms and/or atypical ECG changes. TTE with A-F mnemonic revealed significant cardiac pathology in 127 (46,52%) non-ACS and 23 (2,21%) ACS patients. On the basis of these findings, 20 (1,92%) of ACS and 29 (10,62%) of non-ACS group patients underwent target invasive treatment (aortic valve replacement, mitral annuloplasty, aortic alloplasty, decompression of tamponade, VSD closure, fibrinolysis, removal of infected pacemaker electrode).

All echocardiographic examinations were repeated within 24 hours by a cardiology consultant with expertise in echocardiography, and all digital clips recorded and described based on A-F mnemonic by residents, were reviewed by investigators blinded to the final diagnosis. Comparison of both examinations confirmed 100% coincidence of A-F-based findings. In almost 20% of cases cardiologists reported extra findings extending beyond the A-F scheme, but none of these however had any impact on the choice of the treatment. This excellent coherence between TTE reports done by residents and cardiologists-echocardiographers is puzzling. It may result from their competency in echocardiography. All residents had basic skills in echocardiography with at least 75 exams performed till the end of 2012 (mean 86 examinations/resident).

Vignon et al. [15] in a prospective descriptive clinical study assessed the efficacy of limited tailored echocardiographic training for non-cardiology residents without any ultrasound experience. The authors proved that a 12-hour training program was sufficient for reaching competence in basic critical care echocardiography. Mjolstad et al. [16] examined the feasibility and reliability of pocket-size hand-held echocardiography (PHHE) by medical residents with limited experience in ultrasound. Based on high correlation with reference method, authors concluded that focused examinations performed by residents after a training period, bring a reliable information and have the potential to improve in-hospital diagnostic procedures. Fredericksen et al. [17] compared the diagnostic accuracy of point-of-care (POC) echocardiography performed by a novices versus experienced cardiologists with an expertise in echocardiography, with regard to the assessment of six cardiac conditions: pericardial effusion, left ventricular (LV) dilatation, right ventricular dilatation, LV hypertrophy, LV failure and aortic stenosis. This study showed that a novice examiner was able to detect common and significant heart pathology in six different categories with good accuracy. Muniz Pazeli et al. [18] investigated whether a nephrologist with limited ultrasound training could accurately assess the volume status by inferior vena cava diameter in hemodialysis patients. The inter-examiner agreement between nephrologist and cardiologist was excellent.

Defined competence level is required to perform trustworthy examination. According to European Association of Echocardiography (EAE) [19] 350 examinations must be performed to achieve a basic level of competence. All residents participated in our study apart from a standard TTE training underwent a 1,5-hour structured hands-on dedicated practice with the A-F scheme practical implementation. Survey showed that both echocardiographic image acquisition and interpretation based on A-F mnemonic, usually took less than 5 minutes. All residents found A-F mnemonic algorithm very simple and useful in sticking to the examination structure and helpful in avoiding omissions of important findings.

It is well documented that focused bedside echocardiography can bring important anatomical and hemodynamic information in an acute setting. It can affect the patient’s management, direct further diagnosis and modify therapeutic decision. Panaoulas et al. [20] proved a significant impact of point-of-care (POC) echocardiography on final clinical diagnosis in cardiac patients. Final-year medical students and junior doctors without prior ultrasound experience participated in the study after a 2-hours bedside tutorial. The use of POC echocardiography significantly improved the diagnostic accuracy in comparison to physical examination, medical history and ECG findings. Spencer et al. [21] also noted that POC echocardiography substantially improved the detection of important cardiovascular pathologies compared with physical examination. Manasia et al. [22] reported that limited TTE performed by intensivists, provided new information and altered further management in a significant number of intensive care patients.

We recently validated the usefulness of A-F mnemonic in patients with non-ST-segment elevation ACS [23]. Analysis of 916 patients with suspected NSTE-ACS showed a strong correlation between echocardiographic abnormalities in points B-F of A-F mnemonic with other than coronary ethology of chest pain.

In summary we conclude that: 1. Limited echocardiography with A-F mnemonic is useful in patients with suspected acute coronary syndrome. It allows both confirmation of acute myocardial ischemia and detection of the other life-threatening cardiac conditions requiring definite treatment. 2. This 5 minutes exam covers a sufficient spectrum of morphological and functional abnormalities of the heart, great vessels and adjacent structures, which can alter the diagnostic and therapeutic management in suspected ACS. 3. Residents with a basic knowledge of echocardiography and short training can perform a reliable initial TTE assessment according to A-F mnemonic

### Study limitations and potential pitfalls

Patients with difficult acoustic window (4.3%) were excluded from final analysis because of un-interpretable ultrasound images.

Cardiology consultants did not repeat the study, but reviewed the stored digital 10-seconds video clips, recorded by residents.

Echocardiographic evidence of new regional wall motion abnormalities is one of the diagnostic criteria for an acute myocardial infarction (only when combined with detection of rise and/or fall of cardiac biomarkers) whereas impaired left ventricular global systolic function is a factor related with a poor prognosis. Detection of RWMAs may be challenging especially for non-echocardiographers. Although detection of post-MI scar usually does not cause difficulties (end-diastolic wall thickness < 5 mm, hyperechogenicity), differentiation between preexisting and new hypokinesia may be difficult. Therefore it must be stressed out that echocardiography is only an imaging modality used in the diagnostic process among the others and does not replace clinical reasoning.
